# Pirt Together with TRPV1 Is Involved in the Regulation of Neuropathic Pain

**DOI:** 10.1155/2018/4861491

**Published:** 2018-04-02

**Authors:** Changming Wang, Leying Gu, Yonglan Ruan, Tana Gegen, Lei Yu, Chan Zhu, Yan Yang, Yuan Zhou, Guang Yu, Zongxiang Tang

**Affiliations:** ^1^School of Medicine and Life Sciences, Nanjing University of Chinese Medicine, 138 Xianlin Rd, Nanjing, Jiangsu 210023, China; ^2^Key Laboratory of Chinese Medicine for Prevention and Treatment of Neurological Diseases, Nanjing University of Chinese Medicine, 138 Xianlin Rd, Nanjing, Jiangsu 210023, China; ^3^State Key Laboratory Cultivation Base for TCM Quality and Efficacy, Nanjing University of Chinese Medicine, Nanjing 210023, China; ^4^Key Laboratory of Drug Target and Drug for Degenerative Disease of Jiangsu Province, Nanjing University of Chinese Medicine, Nanjing 210023, China; ^5^Jiangsu Key Laboratory for Functional Substance of Chinese Medicine, Nanjing University of Chinese Medicine, Nanjing 210023, China

## Abstract

Neuropathic pain is a chronic pain and reduces the life quality of patients substantially. Transient receptor potential vanilloid channel 1 (TRPV1), a nonselective cation channel, has been shown to play a crucial role in neuropathic pain. Although TRPV1 plays an important role in neuropathic pain, the mechanism of how TRPV1 was regulated in neuropathic pain remains unclear. Pirt is a membrane protein and binds to TRPV1 to enhance its activity. It was suggested that Pirt should also be involved in neuropathic pain. In this study, we investigated the role of Pirt in neuropathic pain (CCI model); the results show that mechanical allodynia and thermal hyperalgesia were alleviated in *Pirt^−/−^* mice in CCI models. TRPV1 expression was increased by immunofluorescence and real-time PCR experiments. The increase in TRPV1 expression was less in Pirt knockout mice in CCI models. Moreover, the number of capsaicin-responding neurons and the magnitude of evoked calcium response were attenuated in DRG neurons from *Pirt^−/−^* mice in CCI models. Finally, we found that the pain behavior attenuated in dysfunction of both Pirt and TRPV1 was much stronger than in dysfunction of Pirt or TRPV1 only in a CCI model in vitro study. Taken together, Pirt together with TRPV1 is involved in CCI-induced neuropathic pain.

## 1. Introduction

Neuropathic pain is defined as pain arising as a direct consequence of a lesion or disease that affects the somatosensory system at either peripheral or central level [1–3]. Neuropathic pain is a chronic pain which substantially reduced the life quality of patients. 6.9% to 10% of the population is suffering from neuropathic pain worldwide [4], and the number is increasing. Most patients did not achieve adequate pain relief for the current medication due to the dim and complicated mechanism of neuropathic pain [5]. Thus, the study on the mechanism of neuropathic pain is still a huge challenge to the researchers.

One mechanism that has been supposed to modulate neuropathic pain is the transient receptor potential (TRP) channel which detects noxious, irritant, and inflammatory stimuli [6]. Of all these channels, TRPV1 has long been a focus for concern. TRPV1 is the receptor of capsaicin which is important for the detection of noxious heat and low pH [7–9]. Researchers reported that the TRPV1 expression is elevated at both mRNA and protein levels in CCI models in predominantly small-to-medium DRG neurons [10]. The sensitization of TRPV1 is enhanced and involved in the development of mechanical allodynia in neuropathic pain [11]. The modulation of TRPV1 in neuropathic pain was under attention. Acid solution is a suitable medium for introducing a sodium channel blocker through TRPV1 channels to produce a sensory-specific analgesic effect [12]. A full inhibition of the spinal endocannabinoid receptor may lead to TRPV1-mediated analgesic effects in neuropathic pain [13]. By increasing the expression of interleukins and kinases, TRPV1 was sensitized to be involved in the development of neuropathic pain [14]. The modulation of TRPV1 plays an important role in the development and maintenance of neuropathic pain in CCI-induced chronic pain.

The phosphoinositide-interacting regulator of TRP (Pirt) is a transmembrane protein that is expressed in most nociceptive neurons in the dorsal root ganglia (DRG) in the peripheral nerve system (PNS). Pirt has recently been identified as a key modulator of TRPV1 and acts as an endogenous enhancer of TRPV1 function [15]. Considering the crucial role of TRPV1 in neuropathic pain, we hypothesized that Pirt may also play an important role in the development and maintenance of neuropathic pain in CCI models.

## 2. Methods

### 2.1. Animals

This study was approved by the Animal Care and Use Committee of Nanjing University of Chinese Medicine (Nanjing, China). Experiments were conducted according to the animal research ethical guidelines of the International Association for the Study of Pain. Male C57BL/6 mice were housed in groups of four per cage in our GLP animal center, with free access to food and water. For the generation of *Pirt^−/−^* mice, the entire Pirt coding region was replaced with an EGFPf-IRESrtTA-ACN-targeting construct to produce a null allele [15]. Offspring *Pirt^−/−^* and *WT* littermates were generated by breeding heterozygotes. Offspring *Pirt^+/−^* mice were generated by *Pirt^−/−^* and *WT* mice. Only healthy animals weighing 15–20 g and displaying normal water and food intake were included in the study.

### 2.2. Chronic Constriction Injury Model

Mice were anaesthetized with 1% sodium pentobarbital (40 mg/kg, Merck, Darmstadt, Germany) and restrained in a lateral position. A surgical incision was made at the thigh root midline, and the right sciatic nerve was exposed at the middle of the thigh. Three ligatures were tied around the nerve using a 4-0 silk braided cord (Shanghai Pudong Jinhuan Medical Products Co., Ltd.) with a 1 mm space [3]. The force of ligation was based on leg reflex. After the operation, the wounds were sutured shut and the animals were positioned supine during injection and kept in that position until they recovered from anesthesia. A sham operation was performed in the same manner except for sciatic nerve ligation. All surgical procedures were performed under sterile conditions.

### 2.3. Behavior Analysis

As nerve injury leads to mechanical allodynia and thermal hyperalgesia, animal pain behaviors were analyzed. Animal behavior experiments were performed in a controlled environment of 20–24°C, 45–65% humidity, and a 12 h day/night cycle. Male *WT* and *Pirt^−/−^* mice (5–6 weeks old) were used in all experiments. Animals were acclimated to the testing environment for 30 min before the initiation of behavior tests. Animal behaviors were analyzed by investigators who were blind to animal treatment conditions.

### 2.4. Measurement of Thermal Hyperalgesia

Thermal hyperalgesia was assessed by measuring the paw withdrawal latency to radiant heat stimuli after the Von-Frey tests. Mice were placed in elevated chambers on a Plexiglas floor and were acclimated to the testing environment for 30 minutes before the experiments. The radiant heat source (Ugo Basile Plantar Test 37370) was applied to the center of the plantar surface of the hind paw with at least 3 min intervals. The average withdrawal latency of the trials was recorded as the response latency.

### 2.5. Measurement of Mechanical Allodynia

Animals were acclimated to the testing environment for 30 minutes before the initiation of behavioral tests. Animal behaviors were analyzed by investigators who were blind to the grouping. Mechanical allodynia was assessed by measuring the paw withdrawal threshold with a set of Aesthesio Von Frey filaments (0.04–2 g). Mice were placed on an elevated metal grid (100 cm × 50 cm). The filament was applied to the plantar surface at a vertical angle for up to 3 s from the bottom. Fifty percent withdrawal threshold values were determined using the up-down method [16].

### 2.6. Immunostaining of DRG Neurons

DRG tissues were collected from spine levels L4-L5. Pirt expression in *Pirt*^−/−^ mice was evaluated by assessment of the fluorescence of a fused in-frame EGFP fluorescence of the gene [15, 17]. For immunostaining of transient receptor potential cation channel V1 (TRPV1), sections were incubated in blocking solution (containing 3% fetal bovine serum, 0.1% Triton X-100, and 0.02% sodium azide in PBS) for 2 h at room temperature and then with rabbit anti-TRPV1 (1 : 100, Abcam, ab62053) at 4°C overnight. Next, the sections were incubated in Alexa Fluor-conjugated goat anti-rabbit IgG secondary antibody (H + L, 1 : 100, Beyotime, A0453) at room temperature for 2 h. To calculate the positive cells in DRGs, every two slices were captured and counted. The number of fluorescence-positive DRG neurons in L4 and L5 was counted and calculated. After stained pictures were captured, they were merged into an image. Three mice from each group were analyzed. Quantification of immunoreactivity was finished according to the paper published previously [17–19].

### 2.7. Real-Time PCR

Total RNA was extracted from freshly isolated DRGs (L4-L5, S1-S2) using TRIzol reagent (Invitrogen) and was treated with RQ1 DNase (Promega) as before [17, 19]. Reverse transcription was performed using the Transcriptor First Strand cDNA Synthesis kit (Roche, Basel, Switzerland).

For qPCR, Light Cycler 480 SYBR Green I Master (Roche, Basel, Switzerland) was used. The reaction was run in a Light Cycler 480 II Real-Time PCR instrument (Roche, Basel, Switzerland) using 1 *μ*L of the cDNA in a 20 *μ*L reaction according to the manufacturer's instructions. The sequences of the mouse Pirt primers were as follows: forward primer: TAGACGAGAGGTCTCCAGAGT and reverse primer: CCAGTTGCTTTTGGGTGTGG. The sequences of the mouse TRPV1 primers were as follows: forward primer: ATCATCAACGAGGACCCAGG and reverse primer: TGCTATGCCTATCTCGAGTGC. The sequences of mouse GAPDH primers were as follows: forward primer: ACCACAGTCCATGCCATCAC and reverse primer: TCCACCACCCTGTTGCTGTA. Calibrations and normalizations were done using the following 2^−ΔΔCT^ method, where ΔΔCT = (CT (target gene) − CT (reference gene)) − (CT (calibrator) − CT (reference gene)). GAPDH was used as the reference gene for qPCR experiments.

### 2.8. Cell Culture

The mice were anesthetized with 1% sodium pentobarbital. The DRGs were harvested as previously described [17, 19] and immediately transferred to a cold DH10 medium (DMEM/F-12, 10% FBS, and 1% penicillin-streptomycin-glutamine; Invitrogen). DRGs were washed 2-3 times in warm DH10 and then treated with enzyme solution (5 mg/mL dispase and 1 mg/mL collagenase type I in Hanks Balanced Salt Solution without Ca^2+^ and Mg^2+^; Invitrogen) at 37°C until the cells were dissociated [15, 20]. Cell suspensions were then filtered through a 100 *μ*m cell strainer (BD, Franklin Lakes, NJ, USA). After being centrifuged at 1000 rpm for 5 min, DRG neurons were resuspended in DH10, and glial cell-derived neurotrophic factor (GDNF) was added (50 ng/mL, Millipore, Billerica, MA, USA). Fifty microliters of suspended cells in solution was plated onto presterilized glass coverslips that had been coated with 0.5 mg/mL poly-D-lysine (Biomedical Technologies Inc., Stoughton, MA, USA) and 10 *μ*g/mL laminin (Invitrogen). Plated neurons were cultured in an incubator (95% O_2_ and 5% CO_2_) at 37°C and used for calcium imaging studies within 48 h.

### 2.9. Calcium Imaging

The DRG neurons were loaded with fura-2-acetomethoxyl ester (Molecular Probes, Eugene, OR, USA) for 30 min at 37°C in the dark in accord with previous studies [14, 17]. After being washed 3 times with PBS, the glass coverslips were placed into a chamber and perfused with a solution containing 137 mM NaCl, 5.4 mM KCl, 1.2 mM MgCl_2_, 1.2 mM NaH_2_PO_4_, 1 mM CaCl_2_, 10 mM glucose, and 20 mM HEPES (pH 7.4). A high-speed continuously scanning monochromatic light source (Polychrome V, TILL Photonics, Gräfeling, Germany) was used for excitation at 340 and 380 nm, enabling us to detect changes in intracellular free calcium concentration.

### 2.10. Intraperitoneal Injection of AMG9810

*Pirt^−/−^* or *WT* mice in CCI treated or untreated groups underwent intraperitoneal injection of AMG9810 (Sigma-Aldrich, St. Louis, Missouri, United States; TRPV1 antagonist, 3 mg/kg) or vehicle as before [21, 22]. 30 minutes later, the mouse was used for behavior tests.

### 2.11. Data Analysis

All data are presented as mean ± SEM. The number of animals used in each study was based on our experiences and on similar studies. The genotype of mice was blinded to experimenters to reduce selection and observation bias. After the experiments were completed, no data point was excluded. Two-way repeated-measures ANOVA (SPSS version 16.0) was used to compare the behavioral results. For morphological results, we used representative data from three mice with similar results. Statistical analyses of the number of Pirt-positive cells and TRPV1-positive cells were carried out manually. We used representative data from Ca^2+^ imaging studies that were replicated at least 15 times from 3 mice (5 times in each mouse). We used 2-tailed Student's *t*-tests (SPSS version 16.0) to calculate the proportion of cells that responded and the intensity of the response. *P* < 0.05 was considered statistically significant in all tests.

## 3. Results

### 3.1. Mechanical Allodynia and Thermal Hyperalgesia Reduced in Pirt Knockout Mouse in CCI Models

**Figure 1:**
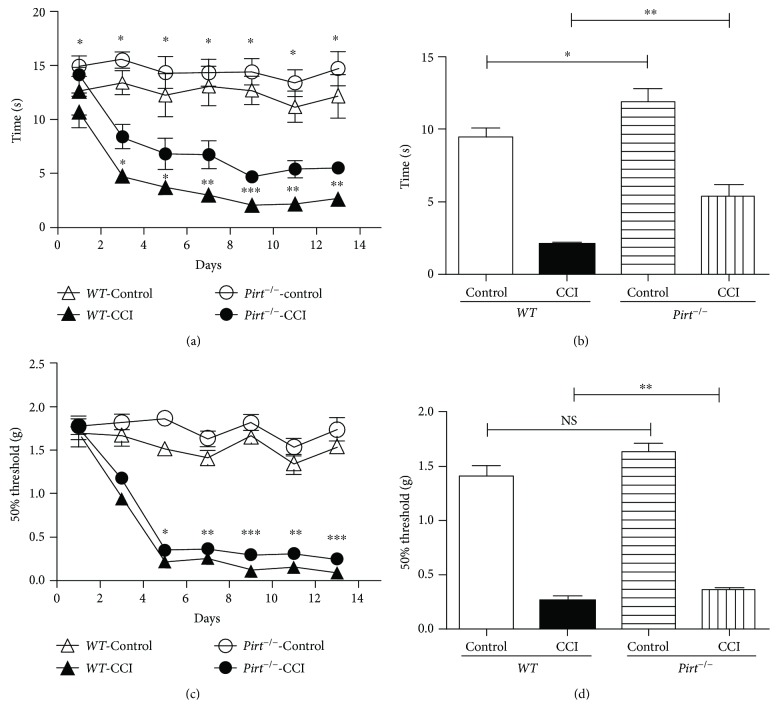
Mechanical allodynia and thremal hyperalgesia behavior reduced in *Pirt^−/−^* mouse in CCI models. (a) Hot plate testing over 14 days on WT and *Pirt^−/−^* mice after CCI or sham operation (control), respectively. Values represent the changes from the baseline TWL values that were recorded on day 0 after operation. (b) The TWL values on the 7th day after the operation were compared. (c) Von-Frey testing over 14 days after CCI or sham operation. Values represent the change from the baseline MWT values that were recorded on day 0 after operation. (d) The MWT values on the 7th day after the operation were compared. ^∗^*P* < 0.05, ^∗∗^*P* < 0.01, and ^∗∗∗^*P* < 0.001.

To determine whether Pirt participates in CCI-induced neuropathic pain, we administered a CCI operation or sham operation to *Pirt^−/−^* mice and *WT* mice, respectively, and then recorded the thermal withdrawal latency (TWL) and the mechanical withdrawal threshold (MWT) on the 1st, 3rd, 5th, 7th, 10th, and 13th days after operation. We found that *Pirt^−/−^* mice respond less to heat stimuli compared with *WT* mice without operation (Figure 1(a)), similar to past research [15]; however, the difference was larger in CCI models especially after the 7th day of operation (Figure 1(b)). The results indicated that Pirt participated in thermal hyperalgesia in CCI models. We did not observe any statistical differences in the MWT values between *Pirt^−/−^* and *WT* mice with the sham operation. By contrast, after the CCI operation, the MWT values of *WT* mice were significantly lower than those of *Pirt^−/−^* mice (Figures 1(c) and 1(d)), suggesting that Pirt participates in mechanical allodynia in the CCI model. These observations suggested that Pirt is involved in maintaining mechanical pain and thermal hyperalgesia in CCI-induced neuropathic pain.

### 3.2. Pirt Together with TRPV1 Modulate Neuropathic Pain in CCI Models

**Figure 2:**
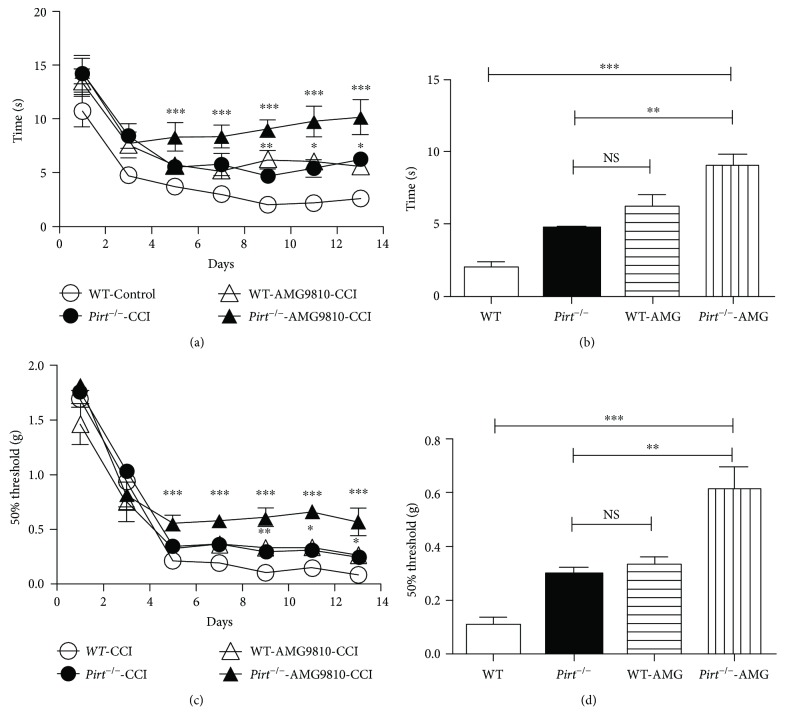
Nociceptive sensation attenuated in dysfunction of both Pirt and TRPV1 in CCI models. (a) Hot plate testing over 14 days on WT, *Pirt^−/−^* mouse, and WT mouse intraperitoneal injection of AMG9810 and *Pirt^−/−^* mouse intraperitoneal injection AMG9810 after CCI operation (control), respectively. Values represent the changes from the baseline TWL values that were recorded on day 0 after operation. (b) The TWL values on the 9th day after the operation were compared. (c) Von-Frey testing over 14 days on WT and WT mouse intraperitoneal injection of AMG9810 and *TRPV1^−/−^* mouse and *Pirt^−/−^* mouse intraperitoneal injection of AMG9810 after CCI operation. Values represent the change from the baseline MWT values that were recorded on day 0 after operation. (d) The MWT values on the 9th day after the operation were compared. ^∗^*P* < 0.05, ^∗∗^*P* < 0.01, and ^∗∗∗^*P* < 0.001.

Pirt has been identified as a key modulator of TRPV1 and acts as an endogenous enhancer of TRPV1 function [15]. To investigate the relationship of Pirt and TRPV1 in CCI models, dysfunction of TRPV1 and Pirt to detect the behavior difference in CCI models was administered. Mechanical allodynia was compared in WT mouse, Pirt knockout mouse, and WT mouse by intraperitoneal injection of AMG9810 (TRPV1 antagonist) and in Pirt knockout mouse by intraperitoneal injection of AMG9810 in CCI models (Figure 2(a)). We recorded TWL and MWT on the 1st, 3rd, 5th, 7th, 10th, and 13th days after operation. We found that *Pirt^−/−^* mice respond differently to heat stimuli compared with *WT* mice in the CCI operation (Figures 2(a) and 2(b), *P* < 0.01, *n* = 8). There was no difference between *Pirt^−/−^* mice and *WT* mice (intraperitoneal injection of AMG9810) in heat stimuli in CCI models. However, TWL in the intraperitoneal injection of AMG9810 in the *Pirt^−/−^* mouse group was enhanced compared with *WT* mice (intraperitoneal injection of AMG9810) on the 10th day. Similarly, *Pirt^−/−^* mice respond differently to mechanical stimuli compared with *WT* mice in the CCI operation (Figures 2(c) and 2(d), *P* < 0.01, *n* = 8). There was no difference between *Pirt^−/−^* mice and *WT* mice (intraperitoneal injection of AMG9810) in heat stimuli in CCI models. However, MWT in the intraperitoneal injection of AMG9810 in the *Pirt^−/−^* mouse group was enhanced compared with *Pirt^−/−^* mice and *WT* mice (intraperitoneal injection of AMG9810) on the 10th day (*P* < 0.01, *n* = 8). In a word, there was no difference in dysfunction of Pirt or TRPV1 only (*Pirt^−/−^* mouse versus *WT* mouse intraperitoneal injection of AMG9810, *P* < 0.01, *n* = 8). However, dysfunction of both Pirt and TRPV1 attenuated the pain behavior compared with dysfunction of Pirt or TRPV1 only. The results indicated that Pirt together with TRPV1 modulates neuropathic pain in CCI models.

### 3.3. TRPV1 Expression Increased in CCI Models

**Figure 3:**
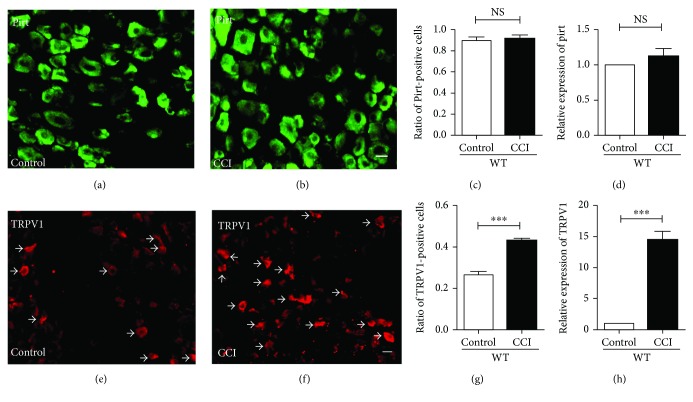
Comparison of expression of Pirt and TRPV1 in DRG in CCI models. Pirt-positive neurons in DRGs in control (a) and CCI (b) groups. (c) Pirt-positive neurons in DRGs compared between control and CCI groups. (d) Real-time PCR results indicate that the expression of Pirt was unchanged in CCI models. Histochemistry staining of DRG from control groups (e) and CCI (f) models. The arrows indicate TRPV1-positive DRG neurons. Scale bar, 20 *μ*m. *n* = 3/group. The fraction of TRPV1-positive neurons in DRG is significantly decreased in CCI models (g). (h) Real-time PCR results indicate that the expression of TRPV1 increased in CCI models. ^∗∗∗^*P* < 0.001

To investigate if the mechanisms of Pirt and TRPV1 modulate neuropathic pain, Pirt and TRPV1 expression in DRGs was studied in CCI models. In *Pirt^−/−^* mice, the coding region of *Pirt* was replaced with an *EGFP* gene as described in our previous study [15]. Taking advantage of EGFP, the DRG neurons specifically expressing Pirt can be visualized by imaging GFP fluorescence in *Pirt^−/−^* mice. As shown in Figures 3(a) and 3(b), in comparison with DRGs from control *Pirt^−/−^* mice, the number of Pirt-positive neurons was unchanged in DRGs from CCI-operated mice (Figure 3(c), CCI versus control, *n* = 3). Consistently, the expressions of *Pirt* at the transcription level in the CCI group were also unchanged as determined by real-time PCR (Figure 3(d)) (*P* < 0.05, *n* = 3). These observations indicated that Pirt expression was unchanged in the CCI operation in DRG neurons. Considering that Pirt may act as a regulatory subunit of TRPV1 in CCI models, TRPV1 expression was detected in WT mice in CCI models by histochemistry staining of DRGs (Figures 3(e) and 3(f)). TRPV1-positive neurons increased substantially in DRGs in CCI models (Figure 3(g)). Identically, the expressions of TRPV1 at the transcription level in the CCI group were enhanced as determined by real-time PCR (Figure 3(d)) (*P* < 0.05, *n* = 3). These observations indicate that TRPV1 expression increased in the CCI operation in DRG neurons while Pirt expression was unchanged.

### 3.4. The Increasing Expression of TRPV1 in Pirt Knockout Mouse Was Less Than in WT Mouse in the CCI Treated Group

**Figure 4:**
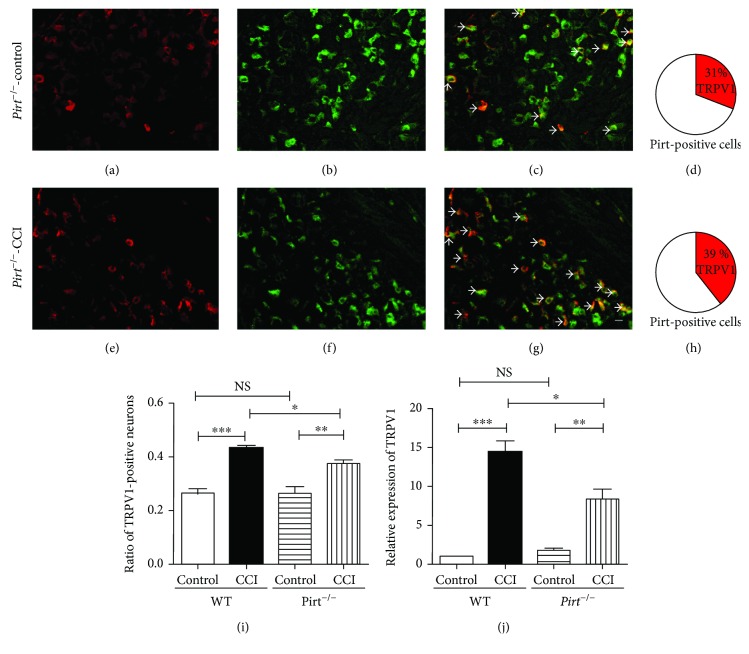
Expression of TRPV1 increased less in Pirt knockout mice in CCI models. Representative images show TRPV1-positive cells (a), Pirt-positive cells (b), or a merge of TRPV1-positive and Pirt-positive neurons (c) in the control group. 31% TRPV1-positive DRG neurons are Pirt positive (d). In CCI models, representative images show TRPV1-positive cells (e), Pirt-positive cells (f), or a merge of TRPV1-positive and Pirt-positive neurons (g) in the control group. 39% TRPV1-positive DRG neurons are Pirt positive (h). The arrows indicate double-labelling cells. Scale bar: 20 *μ*m. The results of histochemistry staining (i) show TRPV1-positive neurons in WT and Pirt knockout mice in CCI models. (j) Real-time PCR results indicate the expression of TRPV1 in WT and Pirt knockout mice in CCI models. ^∗^*P* < 0.05, ^∗∗^*P* < 0.01, and ^∗∗∗^*P* < 0.001.

In nonmodel group mice, TRPV1 expression was unchanged in Pirt knockout mice [15]. To determine the relationship between Pirt and TRPV1 in chronic pain, TRPV1 expression was studied in *Pirt^−/−^* mice in CCI models by two-labeled histochemistry staining (Figures 4(a)–4(h)). The increase in TRPV1-positive neurons was all Pirt positive (Figures 4(d) and 4(h)). We found that TRPV1-positive neurons increased in CCI models in *WT* mice; however, TRPV1-positive neurons increased less in CCI models in *Pirt^−/−^* mice (Figure 4(i), *P* < 0.05, *n* = 3). Consistently, the expression of TRPV1 at the transcription level in *Pirt^−/−^* mice in the CCI group was less than in *WT* mice determined by real-time PCR (Figure 4(j)) (*P* < 0.05, *n* = 3). Meanwhile, the expression of Pirt in TRPV1 knockout mouse in DRGs was detected in the control and CCI-treated groups by real-time PCR. We found that Pirt expression in TRPV1 knockout mouse was unchanged in both control and CCI-treated groups (data not shown). In the results, although Pirt could not change the expression of TRPV1 in nonmodel group mice, the increasing expression of TRPV1 was less in *Pirt^−/−^* mice in CCI models. These observations indicated an important relationship between Pirt and TRPV1 in chronic pain.

### 3.5. Capsaicin-Induced Response Attenuated in Pirt Knockout Mouse in CCI-Treated Sensory Neurons

**Figure 5:**
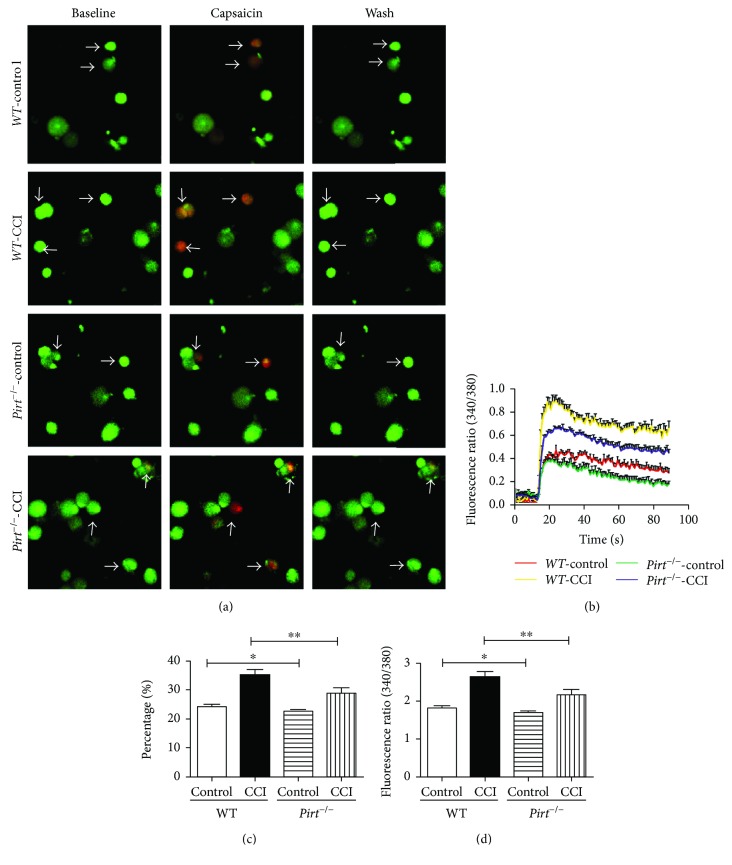
Pirt knockout attenuated capsaicin-induced response in CCI-treated sensory neurons. (a) Fluorescence image of intracellular calcium flux induced by capsaicin. Arrows indicate the response to capsaicin in WT and *Pirt*^−/−^ DRG neurons in CCI models and the control group. Scale bar: 20 *μ*m. (b) Representative fura-2 ratio metric responses in cultured DRG neurons. The curves indicate the response to capsaicin in *Pirt*^−/−^ mice and *WT* mice. (c) The percentages of DRG neurons from WT and *Pirt*^−/−^ mice that responded to capsaicin (% of total neurons, *n* = 3/group). (d) Fluorescence intensities of capsaicin (1 *μ*M)-induced calcium influx. ^∗^*P* < 0.05 and ^∗∗^*P* < 0.01.

To confirm the physiological function of Pirt in CCI models, the capsaicin-induced calcium influx was compared in DRG neurons from *WT* and *Pirt^−/−^* mice in CCI models by calcium imaging (Figures 5(a)–5(d)). The proportion of DRG neurons responding to capsaicin in *WT* mice was significantly higher in CCI models than in controls (Figures 5(a) and 5(c), *P* < 0.001, *n* = 3). There were slight differences in DRG neurons responding to capsaicin between *WT* and *Pirt^−/−^* mice without operation (Figures 5(a) and 5(c), *P* < 0.05, *n* = 3), while a greater difference was presented in *WT* and *Pirt^−/−^* mice in CCI models (Figures 5(a) and 5(c), *P* < 0.01, *n* = 3). The magnitude of capsaicin-evoked calcium response was attenuated in DRG neurons from *Pirt^−/−^* mice in CCI models than from *WT* mice (Figures 5(b) and 5(d), *P* < 0.05, *n* = 3). The attenuation of capsaicin-evoked calcium response in *WT* and *Pirt^−/−^* mice in CCI models indicates that Pirt and TRPV1 modulated neuropathic pain in CCI models.

## 4. Discussion

Neuropathic pain significantly reduced the life quality of patients for severe pain. More importantly, the mechanism of neuropathic pain is still dim. Here, we first used CCI mouse models to show that Pirt together with TRPV1 is involved in CCI-induced neuropathic pain.

Pirt is expressed in most nociceptive DRG neurons. Our past research reported that *Pirt^−/−^* mice show an impaired response to noxious heat stimuli and capsaicin [15]. In our research, both responses to noxious heat stimuli and mechanical stimuli in *Pirt^−/−^* mice declined in CCI-treated mouse. The results indicated that Pirt was more important in response to both noxious heat stimuli and mechanical stimuli in chronic pain conditions.

To illustrate the relationship between Pirt and TRPV1 in neuropathic pain, dysfunction of Pirt and TRPV1 was administered in CCI models. There was no difference in pain sensation between dysfunction of Pirt and dysfunction of TRPV1 in CCI-treated mouse, indicating that both Pirt and TRPV1 were important in the involvement of neuropathic pain. However, dysfunction of both Pirt and TRPV1 attenuated the pain sensation more severely compared with dysfunction of Pirt or TRPV1 only. The results indicated that Pirt together with TRPV1 modulates neuropathic pain in CCI models.

Presently, TRP channels have been known to play a central role in the sensitization of nociceptive transduction in neuropathic pain, such as TRPV1 [6, 10, 14, 23, 24]. Pirt functions as an endogenous regulator of TRPV1 [15]. Therefore, we predicted that Pirt may modulate neuropathic pain by regulation of TRPV1. The response to noxious heat stimuli and mechanical stimuli increased obviously in CCI-treated mouse compared with Pirt knockout mouse. Thus, Pirt may modulate neuropathic pain in CCI models by regulation of TRPV1. The expression and function of TRPV1 were detected in CCI-treated Pirt knockout mice. The expression of Pirt was unchanged while TRPV1 expression increased in CCI models as Pirt is an endogenous regulator of TRPV1 which enhanced the function of TRPV1. In the Pirt knockout mouse, TRPV1 expression was unchanged compared with *WT* mouse in the CCI-untreated group; however, TRPV1 expression increased not so much as in *WT* mouse in CCI models. As a modulator of TRPV1, Pirt could not affect the expression of TRPV1 but affected the increasing expression of TRPV1 in CCI models in neuropathic pain. The DRG neurons respond to capsaicin less in Pirt knockout mouse in CCI models compared with control. In a word, Pirt modulates neuropathic pain by regulation of TRPV1.

Pirt functions as a regulatory subunit of TRPV1 [15]; our past research found Pirt-positive fibers expressed in the uterus and which modulate uterine contraction-induced pain [17]. TRPV1 contributes to both mechanical allodynia and thermal hyperalgesia in past researches [19, 25, 26]. In this study, Pirt together with TRPV1 modulates both mechanical allodynia and thermal hyperalgesia in CCI models in neuropathic pain.

Collectively, we demonstrate that Pirt together with TRPV1 modulates neuropathic pain in CCI models, and highlight a new mechanism for CCI-induced neuropathic pain.

## 5. Conclusion

Our findings indicate that Pirt together with TRPV1 modulates CCI-induced neuropathic pain by comparison of behavior tests in *Pirt^−/−^* mice and *WT* mice and by analysis of the immunohistochemistry and calcium imaging on DRG neurons and dysfunction of Pirt and TRPV1 in CCI models.

## Abbreviations

Pirt: Phosphoinositide-interacting regulator of transient receptor potential
TRPV1: Transient receptor potential vanilloid channel 1
DRG: Dorsal root ganglia
CCI: Mouse model of chronic constriction injury
TWL: Thermal withdrawal latency
MWT: Mechanical withdrawal threshold.
